# *Gossypium hirsutum* gene of unknown function Gohir.A03G007700.1 encodes a potential VAN3-binding protein with a phosphoinositide-binding site

**DOI:** 10.17912/micropub.biology.000669

**Published:** 2023-01-31

**Authors:** Emma R. Smith, Lauryn R. Caulley, Amanda R Storm, Amanda M. Hulse-Kemp, Angela K. Stoeckman

**Affiliations:** 1 Chemistry Department, Bethel University, Saint Paul, MN USA; 2 Department of Biology, Western Carolina University, Cullowhee, NC USA; 3 Genomics and Bioinformatics Research Unit, The Agricultural Research Service of U.S. Department of Agriculture, Raleigh, NC USA; 4 Department of Crop and Soil Sciences, North Carolina State University, Raleigh, NC

## Abstract

A gene of unknown function, Gohir.A03G007700.1 (gene ID: Gohir.A03G007700_UTX-TM1_v2.1; transcript ID: Gohir.A03G007700.1_UTX-TM1_v2.1), identified in *Gossypium hirsutum* was studied using bioinformatic analyses of the sequence and structure of its encoded protein. Results from domain prediction, conserved residues and structure comparison indicate the encoded plant-specific protein (UniProt A0A1U8N485) is part of the VAN3-binding protein family with a conserved phosphoinositide-binding site. Homology comparison suggests functional similarity with Arabidopsis FORKED-like FL5 and 6, which localize to the Golgi apparatus and are linked to vein development and leaf size phenotypes.

**Figure 1. Sequence and Structure Characterization of GhVAB-A0A1U8N485:**
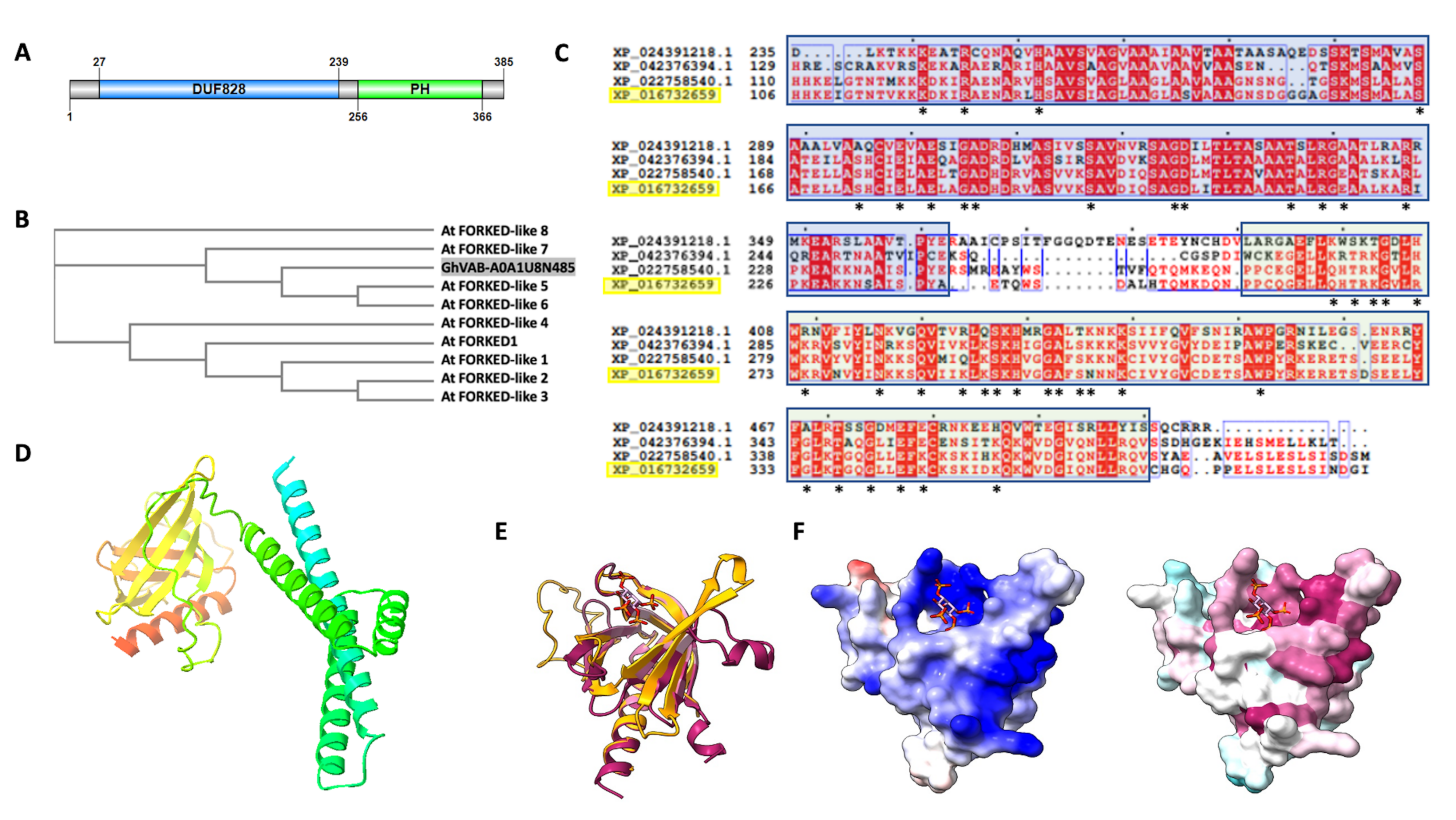
(A) Domain architecture of GhVAB-A0A1U8N485 indicating the location of predicted sequence features. Two primary domains include a domain of unknown function (DUF828) and a pleckstrin homology (PH) domain. Created using Illustrator for Biological Sequences (Liu et al. 2015) based on predictions from InterPro (Blum et al. 2020). (B) Phylogenetic tree of cotton ( *Gossypium hirsutum* ) GhVAB-A0A1U8N485 and *Arabidopsis thaliana* family homologs created using ClustalOmega (Madeira et al. 2019). *Arabidopsis* sequences listed from top to bottom: AT5G57770, AT4G16670, AT4G17350, AT5G47440, AT4G32780, AT3G63300, AT5G43870, AT3G22810, and AT4G14740. (C) Multi-Sequence Alignment of a portion of the DUF828 (blue) and the PH domain (green) showing sequence conservation across diverse homologs. Created using ClustalOmega and ESPript3 (Robert and Gouet 2014). ConSurf-identified highly-conserved functional sequences in GhVAB-A0A1U8N485 indicated with an asterisk (Ashkenazy et al. 2016). Sequences aligned include the following: *Physcomitrium patens* (XP_024391218.1), *Zingiber officinale* (XP_042376394.1), *Durio zibethinus* (XP_022758540.1), and *Gossypium hirsutum* (XP_016732659.1) (highlighted). (D) AlphaFold2 (Jumper et al. 2021; Mirdita et al. 2022) model structure of high confidence region amino acid residues Gly111 through Val366 of GhVAB-A0A1U8N485, with coloration moving from N-terminus (blue) to C-terminus (red). (E) Structure overlay of the GhVAB-A0A1U8N485 PH domain (orange) and human protein kinase B/Akt PH domain bound to phosphatidylinositol (3,4,5)-trisphosphate (pink ribbon and ligand, PDB 1H10). (F) GhVAB-A0A1U8N485 PH domain modeled with ligand showing electrostatic surface calculated by ChimeraX (Version 1.3, Pettersen et al., 2021) (left) and surface coloring based on ConSurf conservation scores (right).

## Description

Introduction

As a top food and fiber crop in the United States, cotton plays a vital role in both the country’s economic health and the day-to-day lives of many individuals. Sequencing the cotton genome provides genetic information not previously available, including genomic structure, variation, and diversity, which can lead to improved cotton sustainability (Peng et al. 2021). Here, we predict the encoded function of an unknown gene following the recent publication of genome sequences from five cotton species (Chen et al. 2020).

According to our findings, the uncharacterized *Gossypium hirsutum* gene Gohir.A03G007700.1 and associated protein (CottonGen: UTX-TM1_v2.1; LOC107943416; UniProt A0A1U8N485; NCBI XP_016732659), here referred to as GhVAB-A0A1U8N485, is a member of the VAN3-binding protein family (IPR040269). A homolog, vascular network defective-3 (VAN3) binding protein (also known as FORKED1; Naramoto et al. 2016), is found in *Arabidopsis thaliana* (AT3G63300). It is proposed that members of the *Arabidopsis* FORKED-like (FL) gene family are involved in aiding asymmetrical localization of the PIN1 (PINFORMED) auxin efflux protein, a process necessary to form closed vein loops and vascular patterns (Hou et al. 2010). The PIN1 efflux protein falls into a family of proteins involved in passive transport of auxin hormones (Singh et al. 2019) and is responsible for establishing vein patterns in leaves. Furthermore, FL genes may be involved in facilitating vein continuity as well as reconciling the number of veins with the size of the leaf (Prabhakaran Mariyamma et al. 2017). Also, based on our analysis, GhVAB-A0A1U8N485 contains a pleckstrin homology (PH) domain and may be regulated by a pathway involving inositol triphosphate. Often localized to membranes, PH domains contain a conserved set of secondary structures and commonly bind phosphatidylinositol phosphates involved in signaling pathways (Le Huray et al. 2022). Indeed, the PH domain was found to be critical in the appropriate subcellular localization of *Arabidopsis* VAN3 (Naramoto et al. 2009).

Sequence Features

The InterPro web server (Blum et al. 2020) identified the 385-amino acid GhVAB-A0A1U8N485 protein as a member of the ‘VAN3-binding protein’ family (IPR040269). The sequence was found to contain a domain of unknown function, DUF828, (IPR008546) [also called an auxin canalisation domain (PF05703)] and a ‘pleckstrin homology (PH) domain’ (IPR001849) ( **Figure 1A)** .

After utilizing the subcellular localization programs SignalP (Almagro Armenteros et al. 2019), TargetP (Almagro Armenteros et al. 2019a), LOCALIZER (Sperschneider et al. 2017), Plant-mSubP (Sahu et al. 2019), BUSCA (Savojardo et al. 2018), YLoc (Briesemeister et al. 2010), and Plant-mPLoc (Chou and Shen 2010), no confident prediction of the subcellular location of GhVAB-A0A1U8N485 was identified. Also, these same subcellular prediction platforms gave conflicting information for the *Arabidopsis* homolog FORKED1 (AT3G63300) suggesting its location, although with low confidence, to be the nucleus, plastid, or mitochondrion. However, experimental evidence demonstrates that FORKED1 (FKD1) localizes to the *trans* -Golgi network (Naramoto et al. 2009). Lastly, we found no indication of transmembrane helices using Phobius (Kall et al, 2007), TMHMM (Krogh et al, 2001), and HMMTOP (Tusnady et al, 2001).

Homology

Using ClustalOmega (Madeira et al. 2019), we generated a phylogenetic tree to show the relationship between GhVAB-A0A1U8N485 and the *Arabidopsis* FL family members ( **Figure 1B** ). GhVAB-A0A1U8N485 appears to be most closely related to FL-5, 6, and 7 which has been considered the Group 3 clade of the *Arabidopsis* family members (Prabhakaran Mariyamma et al. 2018). Experimentally, the Group 3 proteins FL5 and FL6 appear to be primarily localized to the Golgi apparatus whereas the Group 1 proteins (FKD1 and FL1-FL3) localize to the *trans-* Golgi network. Interestingly, mutations in the Group 1 proteins lead to an increase in leaf size and reduction in vein density, while mutations in FL6 and FL7 decreased the size of the leaf.

Using an NCBI BlastP search, homologs of GhVAB-A0A1U8N485 were found only in three categories of the Kingdom Plantae, including bryophytes ( *Physcomitrium patens* (XP_024391218.1)), monocots ( *Zingiber officinale* (XP_042376394.1)), and eudicots ( *Durio zibethinus* (XP_022758540.1)). No homologs were identified in diverse species belonging to animalia, fungi, gymnosperms, ferns, archaebacteria, bacteria, and cyanobacteria. Additionally, all three of the identified homolog proteins were labeled as VAN3-binding proteins. We used ClustalOmega and ESPript3 (Robert and Gouet, 2014) to create a Multi-Sequence Alignment (MSA) ( **Figure 1C)** in order to visualize sequence conservation across the plant categories. There appeared to be conservation of amino acids in both the DUF828 and the PH domain, demonstrating that these regions of our sequence likely remain important across multiple species. Furthermore, many of these residues were indicated by ConSurf (Ashkenazy et al. 2016) to be highly conserved across numerous species as well as functional and are marked with an asterisk. The full ConSurf results for GhVAB-A0A1U8N485 are available as Extended Data.

Structural Features

We used the AlphaFold2 Phenix CoLab (Jumper et al. 2021; Mirdita et al. 2022) to predict a tertiary structure for GhVAB-A0A1U8N485 ( **Figure 1D** ). The program predicted two distinct regions with confident folding in the residue ranges Gly111-Arg224 and Gln259-Val366. The first region, from residues 111-224, consisted primarily of four alpha helices and is within the DUF828 predicted by InterPro. The second region with predicted secondary structure, from the PH domain residues 259-366, contained a single alpha helix and seven anti-parallel beta sheets. Structural evidence of PH domains from the *Saccharomyces cerevisiae* protein Avo1 (PDB 3ULB) and its human homolog Sin1 (3VOQ) demonstrate that at the core of the PH domain is a seven-stranded antiparallel beta-sandwich closed at its C-terminus by an alpha-helix (Pan and Matsuura 2012). At the N-terminus of this beta-sandwich are three variable loops containing positively charged residues forming a pocket of basic residues that bind negatively-charged phosphoinositides. When the PH domain of human protein kinase B/Akt containing the bound phosphatidylinositol trisphosphate ligand (PDB 1H10) was overlaid with the predicted PH domain of GhVAB-A0A1U8N485 ( **Figure 1E** ), a binding pocket accommodating the phosphoinositide ligand was observed in our structure. Indeed, this GhVAB-A0A1U8N485 binding pocket is lined with positively-charged amino acids (blue) ( **Figure 1F left** ) and these residues are highly-conserved (purple) ( **Figure 1F right** ). Mutagenesis of amino acid residues in the standard PH domain fold of human protein kinase B/Akt demonstrates that the phosphates at positions 3 and 4 of phosphatidylinositol (3,4,5)-trisphosphate provide the largest contribution to binding affinity and that amino acid resides K14, R23, R25, N53 and R86 are responsible for this binding (Thomas et al. 2002). A structure-based alignment between GhVAB-A0A1U8N485 and human protein kinase B/Akt using PROMALS3D (Pei et al. 2008) identified Q264, R272, K274, N303, and L342 as the corresponding residues in GhVAB-A0A1U8N485, respectively. Two of these residues are completely conserved, with two others being conservative substitutions. Although a significant difference exists between the properties of R86 and L342, this may reflect a variation in the ability of these PH domains to interact with the phosphate at position 5 of phosphatidylinositol (3,4,5)-trisphosphate, as evidence has suggested that this phosphate plays a key role in binding to the PH domains of Bruton’s tyrosine kinase, dual adaptor for phosphoinositides and phosphotyrosine, and the general receptor for phosphoinositides-1 (Ferguson et al. 2000).

Conclusion

In considering evidence from sequence features and the homology results, we predict that GhVAB-A0A1U8N485 is part of the VAN3-binding protein family. Furthermore, based on functional reports of other VAN3-binding proteins as well as structural analysis of our protein, GhVAB-A0A1U8N485 might be involved in regulating vein development in the leaves of cotton plants by binding phosphoinositides in a signaling pathway. Having an efficient vascular system allows the cotton plant to respond to stresses such as drought and microorganism infection.

## Extended Data

Description: ConSurf sequence conservation results for A0A1U8N485. Resource Type: Dataset. DOI: 10.22002/rf8g5-hm387

Description: ClustalOmega MSA of PhyloGenes phylogenetic tree. Resource Type: Dataset. DOI: 10.22002/dbjn1-37k44
